# Modern Approaches to de novo Synthesis of Extended DNA Fragments: Assembly of a Wide Repertoire of Sequences

**DOI:** 10.32607/actanaturae.27362

**Published:** 2024

**Authors:** T. A. Semashko, G. Y. Fisunov, E. A. Tsoy, D. R. Kharrasov, I. K. Chudinov, D. V. Evsyutina, G. Y. Shevelev, V. M. Govorun

**Affiliations:** Research Institute for Systems Biology and Medicine, Moscow, 117246 Russian Federation; Lopukhin Federal Research and Clinical Center of Physical-Chemical Medicine, Moscow, 119435 Russian Federation; Institute of Chemical Biology and Fundamental Medicine, Novosibirsk, 630090 Russian Federation

**Keywords:** DNA synthesis, DNA artificial synthesis, gene synthesis, gene assembly, PCR assembly, polymerase cyclic assembly, spermidine

## Abstract

The standardization of DNA fragment assembly methods for many tasks of synthetic biology is crucial. This is necessary for synthesizing a wider repertoire of sequences, as well as for further automation and miniaturization of such reactions. In this work, we proposed conditions for the assembly of DNA fragments from chemically synthesized oligonucleotides and we identified the errors occurring in the sequence under these conditions. Additionally, we proposed conditions for further combining synthetic fragments into larger DNA fragments. We showed that the optimized conditions are suitable for the assembly of a wide range of sequences.

## INTRODUCTION

Attempts have been made to obtain synthetic DNA fragments from chemically synthesized short oligonucleotides since the mid-60s of the XX century. However, the assembly of the yeast alanine tRNA gene with a length of 77 bp was successfully completed only in 1970 [1]. The development of techniques for the chemical synthesis of oligonucleotides, as well as the assembly and cloning of extended DNA fragments, currently allows for the creation of entire genomes of viruses [2], prokaryotes [3, 4], and eukaryotes [5], including those designed by researchers – with codon transcoding [6], a four-letter genetic code [7]. Synthetic biology is a rapidly developing field. In many cases, the synthesis of extended DNA sequences is necessary to achieve its ambitious goals of creating organisms with desired properties. The assembly of the whole genome is considered as an achievement of modern technology and is quite an undertaking. In biotechnology, medicine, as well as in fundamental research, the synthesis of *de novo *DNA fragments (containing, as usual, sequences of one or more genes with a length of several kb) with high accuracy and at low cost is of central importance [8], in particular, for heterologous gene expression [9], and their modification [10]. Currently, DNA synthesis *in vitro *is performed hierarchically: first, oligonucleotides are synthesized chemically, and then DNA fragments ranging in size from 0.5 to several kb are assembled from them. If necessary, these fragments are combined with each other to form DNA 2–10 kb long by restriction and ligation, assembly of overlapping fragments, or site-specific recombination [11].

The chemical synthesis of oligonucleotides is an automated, well–rotated process. The main goals are to obtain longer sequences (more than 100 bp), increase the yield of the reaction at all stages of the synthetic cycle, and reduce the number of errors by improving the quality of chemical reagents [12]. Three main strategies for the assembly of oligonucleotides into dsDNA fragments have been developed: *in vitro *assembly using enzymes – ligase cyclic assembly (LCR) [13, 14] and polymerase cyclic assembly (PCR), as well as *in vivo *assembly in yeast cells [15]. The main advantages of PCR assembly are the smaller concentration of the oligonucleotides required for the reaction, the absence of an oligonucleotide phosphorylation stage, and lower labor intensity [16, 17]. Various modifications of PCR have been proposed to assemble long fragments and increase the accuracy of the synthesized sequence [18, 19, 20]. At the same time, given the wide variety of target dsDNAs, determination of the optimal conditions for PCR remains relevant, which include the components of the reaction mixture (buffer system, concentrations of salts, magnesium ions, dNTP, oligonucleotides, type of DNA polymerase, the presence of additives), as well as the temperature and time at each stage of PCR. Amides [21], dimethyl sulfoxide [22], betaine [23], glycerin [24], polyethylene glycols, polyamines [25], in particular spermidine [26], are used as additives. Regardless of the type of assembly being performed, successful synthesis requires a rational design of oligonucleotides that takes into account the thermodynamic characteristics of the sequence and the presence of repeating elements and motifs capable of forming secondary structures. The purpose of this work was to design universal conditions for the assembly of DNA fragments suitable for most such tasks.

In this work, we optimized the PCR assembly of DNA fragments from oligonucleotides and we selected the conditions in which DNA fragments up to 1.5 kb with a diverse repertoire of sequences could be efficiently assembled, taking into account product yield and possible errors in the DNA sequence. We optimized the conditions for combining several amplicons into a fragment of up to 7.5 kb.

## EXPERIMENTAL

**Design and synthesis of oligonucleotides for the assembly of DNA fragments**

The oligonucleotide design was made using the SynthBac program [27, 28] with a thermodynamically optimized method. The synthesis of the oligonucleotides for the 1 000 bp model fragment and the BseRI gene was carried out on the AFM-800 synthesizer (Biosset, Russia), and the synthesis of the oligonucleotides for the transposase gene and fragments of the N4 phage was carried out on the Dr. Oligo 768XLc synthesizer (Biolytic, USA).

**PCR assembly of DNA fragments from the oligonucleotides**

The assembly was carried out in two stages. At the first stage, 2 μl of an oligonucleotide mixture (concentration of 1 000, 100, 10, or 1 nM each) was added to a reaction mixture containing 0.5 μl of polymerase, and a buffer corresponding to the polymerase used; 0.2 mM dNTP (Evrogen, Russia). Additionally, MgSO_4_ (Fermentas, USA) was added to the reaction to a final concentration of 5 or 10 mM; formamide, to a final concentration of 2%; PEG 4 000 (50%) (Fermentas, USA) to a final concentration of 7.5%; or spermidine, to a final concentration of 0.5 or 2.5 mM. Taq polymerases (5 U/μl, Lytech, Russia), Tersus (50X, Evrogen) with commercial buffers, or Phusion obtained in the laboratory with 1× reaction buffer (10 mM Tris-HCl, pH 8.8, 50 mM KCl, 2.5 mM MgSO_4_, 0.1% Triton X-100, 0.2 mg/ml of BSA). The activity of the obtained Phusion DNA polymerase matched the activity of commercial Phusion Hot Start II DNA Polymerase (2 U/μl, Thermo Fisher Scientific). The main program for the assembly was: 95°C, 3 min, then 20 cycles –95°C 30 s, 58°C 30 s and 72°C 1 min, final elongation –72°C 5 min. Other variants of the program were also used in which the temperature gradient was used at the annealing stage (55, 55.9, 57.6, 60.1, 63.2, 65.8, 67.3 or 68°C), 2 minutes of elongation or 30 reaction cycles.

At the second stage, the completed DNA fragment was amplified. After the first stage of PCR, 2 μl of the reaction mixture was transferred to a reaction mixture containing 20 mM Tris-HCl pH 8.8, 10 mM KCl, 2 mM MgSO_4_, 6 mM (NH_4_)_2_SO_4_, 0.1% Triton X-100, 0.1 mg/ml BSA, Phusion DNA polymerase, 250 nM of each primer, 0.2 mM dNTP. PCR program: 95°C 3 min, then 25 cycles –95°C 30 s, 58°C 30 s and 72°C 1 min, final elongation –72°C 5 min.

**The assembly of the transposase gene**

The gene was assembled in two stages. At the first stage, 2 μl of an oligonucleotides mixture (500, 100, 10 or 1 nM each) was added to the reaction mixture with 0.5 μl of polymerase, and a buffer corresponding to the polymerase used, 0.2 mM dNTP. Additionally, 10 mM MgSO_4_ or 2.5 mM spermidine was present in the reaction. Taq polymerases (5 U/μl, Lytech) or Phusion obtained in the laboratory with 1× reaction buffer (10 mM Tris-HCl, pH 8.8, 50 mM KCl, 0.1% Triton X-100, 0.2 mg/ml BSA) were used. The activity of the obtained Phusion DNA polymerase matched the activity of commercial Phusion Hot Start II DNA Polymerase (2 U/μl, Thermo Fisher Scientific). The main program for the assembly was: 98°C 3 min, then 20 cycles –96°C 15 s, 57°C 20 s and 72°C 1 min.

At the second stage, 2 μl of the resulting reaction mixture was transferred to 25 μl of the reaction mixture with 50 mM Tris pH 8.8, 100 mM KCl, 2.5 mM MgSO_4_, 0.1% Triton X-100, 0.2 mg/ml BSA, DNA polymerase Phusion, 300 nM of each primer, 0.2 mM dNTP. PCR program: 96°C 1 min, then 25 cycles –95°C 15 s, 57°C 20 s and 72°C 1 min.

**Error rate calculations in the assembled DNA fragment**

To determine the extent to which the DNA polymerase used affected the frequency of different types of errors in the assembled DNA fragment, the transposase gene was assembled under the conditions described below. Taq (5 U/μl, Lytech) or laboratory- obtained Phusion with 1× reaction buffer (10 mM Tris-HCl, pH 8.8, 50 mM KCl, 0.1% Triton X-100, 0.2 mg/ml BSA) were used as DNA polymerase. The activity of the obtained Phusion DNA polymerase matched the activity of commercial Phusion Hot Start II DNA Polymerase (2 U/μl, Thermo Fisher Scientific). The gene was assembled in two stages. In the first stage, 2 μl of the oligonucleotide mixture was added to the reaction mixture (0.5 μl of polymerase, buffer corresponding to the polymerase used, 0.2 mM dNTP and 2.5 mM spermidine). The main program for assembly was as follows: 98°C 3 min, then 20 cycles –96°C 15 s, 57°C 20 s and 72°C 1 min.

In the second stage, 2 μl of the resulting reaction mixture was transferred to 25 μl of the reaction mixture with 10 mM Tris-HCl pH 8.8, 100 mM KCl, 2.5 mM MgSO_4_, 0.1% Triton X-100, 0.2 mg/ml BSA, DNA polymerase Phusion, 300 nM of each primer, 0.2 mM dNTP. PCR program: 96°C 1 min, then 25 cycles –95°C 15 s, 57°C 20 s and 72°C 1 min.

The gene assembled using Taq DNA polymerase was cloned into the pET15 vector using NEBuilder (NEB, USA) and chemically transformed into *E. coli* Top10 cells. The gene assembled using Phusion DNA polymerase was cloned into the pTZ57RT vector using homologous recombination *in vivo *after chemical transformation into *E. coli *strain Top10 carrying plasmid pKM200 (Addgene) with the Lambda Red recombination system. Some 18 clones of each gene variant were sequenced by the Sanger method on the Honor 1616 genetic analyzer (Nanjing Superyears Gene Technology Co., Ltd., China).

Subsequently, the conditions described above and the laboratory-obtained Phusion DNA polymerase with a 1× reaction buffer were used to assemble DNA fragments up to 1 500 bp long.

**Combining DNA fragments using PCR**

dsDNA fragments with lengths of 1 009 (fragment 1), 1 152 (fragment 2), and 1 254 (fragment 3) bp, obtained after assembly from oligonucleotides, were combined into pairs (2 and 3) and triples (1, 2 and 3). The amount of matrix added to the reaction was varied. When combining a pair of fragments, the concentration of each of them in the reaction mixture was 3 nM, 300 pM, 30 pM, and 3 pM; for triples it was 2 nM, 200 pM, 20 pM, and 2 pM. The concentration of the fragments was measured using a Qubit fluorimeter (Thermo) and a dsDNA BR Assay Kit (Thermo). During the assembly of three fragments, amplicons were introduced both without purification (in the form of a reaction mixture after assembly from oligonucleotides) and purified on magnetic particles NEBNext Sample Purification beads (NEB) according to the manufacturer’s protocol. Each sample contained 0.4 μl of Taq polymerase (5 U/μl, Lytech) or Tersus (50X, Evrogen), an appropriate commercial buffer, 0.2 mM dNTP and a pair of primers with a final concentration of 0.25 μM in the mixture. The amplification reaction was performed at spermidine concentrations of 0, 0.5, and 2.5 mM. Amplification conditions: 95°C 3 min, then 20 cycles –95°C 30 s, 62°C 30 s and 72°C 5 min, final elongation –72°C 5 min.

**Visualization of the assembly of fragments**

Visualization was performed using electrophoretic separation of DNA fragments in a horizontal 1% agarose gel in 0.1 M Tris-borate buffer at 150 V for 20–40 min.

## RESULTS

**Condition optimization for the assembly of DNA fragments from oligonucleotides**

To optimize conditions, we used a fragment of the* Mycoplasma gallisepticum *S6 ribosomal protein operon, consisting of the rpsJ gene and the first half of the rplC gene (1016 bp). Using the SynthBac program developed by our group (manuscript in preparation), the fragment was divided into 47 overlapping thermodynamically optimized oligonucleotides with an average length of 43 bp [27]. The gene was assembled in two PCR stages: in the first stage, the oligonucleotides were extended to assemble the fragment with a complete sequence: in the second stage, when flanked primers were added, the resulting fragment was amplified.

**Fig. 1:**
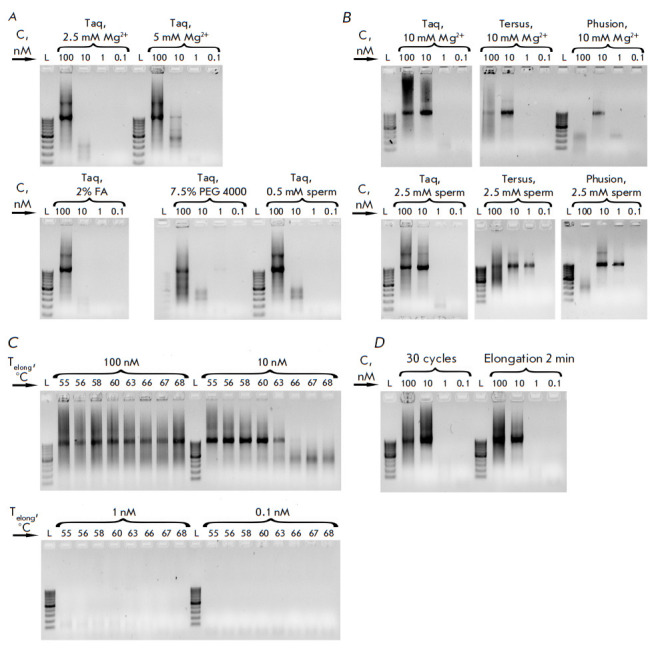
Optimization of assembly conditions for a 1 kb model fragment. (*A*) – assembly of the fragment using additives in the reaction mixture that do not significantly affect the assembly efficiency. (*B*) – assembly of fragments by various polymerases using additives in reaction mixtures that improve the efficiency of assembly. (*C*) – assembly at different annealing temperatures. (*D*) – assembly with variation in the number of cycles or elongation time. L is the length marker GeneRuler 100 bp (Thermo), sperm is spermidine, C – each oligonucleotide concentration in the reaction mixture

In this work, the PCR conditions of the first stage were optimized (*Fig. 1*). All reaction conditions were applied to the oligonucleotides included into the reaction at four different concentrations (100, 10, 1 and 0.1 nM each in the reaction mixture). According to the results obtained, the optimal range of oligonucleotide concentrations for the assembly of DNA fragments is in the region of tens of nM and varies slightly depending on the composition of the reaction mixture. In the reaction mixture, we varied the concentration of Mg^2+^ ions (2.5, 5 and 10 mM) and found that it has a significant effect on the final product – fragments are much better assembled in the presence of 10 mM Mg^2+^, although such high concentrations are no longer used for fragment amplification (*Fig. 1 A,B*). The effect of formamide and PEG 4000 in the reaction mixture on the assembly of fragments has also been studied. The addition of these components does not have a significant effect (*Fig. 1A*). Interestingly, the addition of 2.5 mM spermidine to the reaction mixture significantly improved the assembly of DNA fragments (*Fig. 1B*). An increase in the elongation time or the number of cycles in the assembly program also contributed to a better assembly of the fragments (*Fig. 1D*). At high concentrations of oligonucleotides, the assembly reaction proceeds effectively over a wide range of annealing temperatures; however, with a decrease in concentration, a decrease in hybridization, usual for PCR, is observed with an increase in the annealing temperature (*Fig. 1B*). The effectiveness of PCR assembly was also investigated when using different polymerases – Taq, Tersus and Phusion – under optimal assembly conditions (with 10 mM Mg^2+^ or 2.5 mM spermidine) (*Fig. 1B*). We showed that all the studied polymerases efficiently collect DNA fragments from oligonucleotides, but that they possess different optimal ranges of concentrations of the oligonucleotides used.

**Assembly of the transposase gene**

**Fig. 2:**
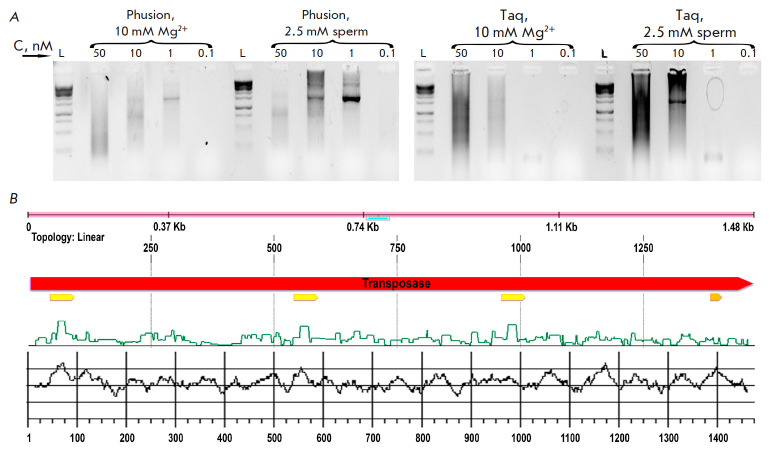
(*A*) – Optimization of transposase gene assembly conditions. L is the length marker GeneRuler 1 kb (Thermo), sperm is spermidine, C – each oligonucleotide concentration in the reaction mixture; (*B*) – Annotation of the transposase sequence in the SynthBac program window. The red arrow indicates the coding frame of the transposase, the yellow one indicates the hairpin, the dark yellow one indicates the motif with a potential G-quadruplex, and the green line indicates the possibility of formation of a secondary structure. The black line on the bottom panel shows the GC composition calculated in the window of 20 bp

The transposase gene (1,476 bp) was divided into 64 oligonucleotides with an average length of 45 bp using the SynthBac program [27] with an algorithm for thermodynamically optimized oligonucleotides. Most of the genes were successfully assembled using the method we optimized, but the transposase gene possesses an arduous sequence to assemble. We experimentally determined that the difficult fragment is located closer to the 3’ end of the gene (data are not provided). Regions of the gene with heterogeneous GC composition, secondary structures such as three identified potential hairpins, or the GGGTGCACTGTGGGAGGGCTGGG motif predicted [29] as a potential G-quadruplex proved difficult to assemble (*Fig. 2B*). We were able to obtain the required fragment (full-size (*Fig. 2A*) or divided into two approximately equal parts) only when carrying out an assembly reaction in the presence of 2.5 mM spermidine. Thus, spermidine also increases the specificity of the reaction.

**Fig. 3:**
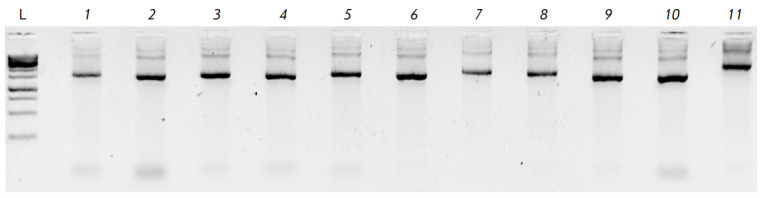
Validation of the proposed method and conditions for DNA assembly from oligonucleotides on 11 different sequences with a length of approximately 1 500 bp

The universality of the proposed conditions for the assembly of various sequences was confirmed by successfully assembling 11 different DNA fragments with a length of about 1.5 kb when adding spermidine to the reaction mixture at the first stage of the assembly (*Fig. 3*).

**Table 1:** Error rates per 1 kb when assembling the transposase gene using Taq or Phusion DNA polymerase^*^

Type of error	Assembly using Taq DNA polymerase	Assembly using Phusion DNA polymerase
Substitutions	5.95 ± 0.47	0.90 ± 0.18
Transitions
	G/C to A/T	3.58 ± 0.48	0.26 ± 0.10
	A/T to G/C	0.98 ± 0.16	0.08 ± 0.05
Transversions
	G/C to C/G	0.30 ± 0.13	0.19 ± 0.07
	G/C to T/A	0.30 ± 0.13	0.19 ± 0.07
A/T to C/G	0.30 ± 0.11	0.11 ± 0.06
A/T to T/A	0.49 ± 0.11	0.08 ± 0.05
Deletions
	Single base	1.17 ± 0.16	1.09 ± 0.22
	Multiple bases	0.41 ± 0.15	0.30 ± 0.11
Insertions
	Single base	0.56 ± 0.15	0.49 ± 0.14
	Multiple bases	0	0.19 ± 0.07
Total errors	8.09 ± 0.66	2.97 ± 0.30

^*^The data is presented as mean value for 18 independent samples with a standard error.

**Error rate in the DNA sequence identification resulting from the assembly of fragments**

We studied the effect of the DNA polymerase type used at the first stage of DNA fragment assembly on the number of various types of errors in the final assembly of the target fragment. (*Table 1*). We demonstrated that the assembly with Taq polymerase yields fragments with an error rate of 8 per 1 kb; and the assembly with Phusion polymerase – three errors per 1 kb (total errors in *Table 1*). At the same time, the frequencies of insertions and deletions occurring in fragments were a match, and the main difference when using different polymerases was the number of substitutions, especially the G/C transitions in A/T.

**Combining several DNA fragments using PCR**

**Fig. 4:**
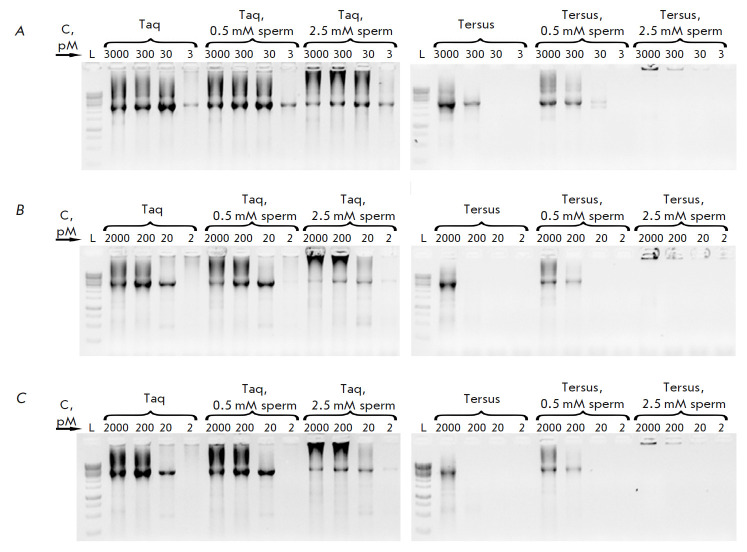
Optimization of the conditions for combining fragments of the BseRI gene. L is the length marker GeneRuler 1 kb (Thermo), sperm is spermidine, C – each oligonucleotide concentration in the reaction mixture. (*A*) – combining two fragments, 2 and 3; (*B*) – combining three fragments, 1, 2, and 3; (*C*) – combining three previously purified fragments, 1, 2, and 3

The BseRI restriction endonuclease gene with a length of 3 348 bp was divided into three overlapping fragments with lengths of 1 009 (fragment 1), 1 152 (fragment 2), and 1 254 bp (fragment 3). Each of the fragments was also divided into oligonucleotides using the SynthBac program [27] with an algorithm for thermodynamically optimized oligonucleotides and assembled according to the method optimized by us. The fragments were combined in two (*Fig. 4A*) and three fragments in one reaction (*Fig. 4 B,C*). The reaction of combining fragments after preliminary purification was also performed (*Fig. 4B*). In all the selected variants, a full-size product with approximately the same efficiency was obtained. Various reaction conditions were analyzed, such as the assembly with Taq or Tersus polymerase, different concentrations of oligonucleotides, as well as the addition of 0.5 or 2.5 mM of spermidine to the reaction. In this case, the reaction substrates had optimum concentrations of tens of pM for Taq polymerase and hundreds of pM for Tersus, and spermidine did not optimize the reaction.

**Fig. 5:**
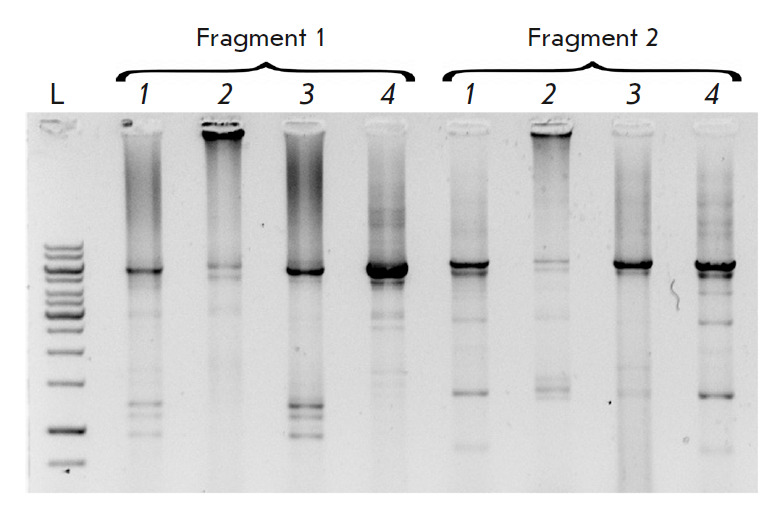
Optimization of the conditions for combining five 1.5 kb fragments into one. Fragment 1 and fragment 2 are different fragments of vibriophage N4, L is the length marker GeneRuler 1 kb (Thermo), 1 is the usual conditions of combining PCR, 2 is PCR with the addition of 2.5 mM spermidine, 3 is PCR after the Gibson reaction, and 4 is PCR with elongation at 65°C and extended elongation time

The genome DNA of vibriophage N4, 38.5 bp long, was divided into fragments about 1.5 kb long using the SynthBac program [27]. Each such fragment was pre-assembled from oligonucleotides using a method optimized by us. It was not possible to assemble longer fragments of up to 7.5 kb from five fragments using a technique optimized for smaller fragments; however, a decrease in temperature and an increase in elongation time allowed us to reproducibly assemble DNA fragments of up to 7.5 kb (*Fig. 5*). Fragments of similar length can also be achieved by amplifying the matrix after the Gibson reaction [30].

## DISCUSSION

We have chosen the PCR assembly method from the most commonly used methods of DNA assembly from oligonucleotides. The advantages of the PCR assembly method in comparison with ligase-cyclic assembly (LCR) are a lower concentration of oligonucleotides in the reaction, fewer assembly stages, and the use of only DNA polymerase.

The assembly of DNA fragments by PCR consists of two stages. In the first stage, the oligonucleotides hybridize with each other and are extended to form the required fragment, and in the second stage, the fullsize fragment is amplified. In this work, the first stage of PCR assembly is optimized – the stage of elongation of the oligonucleotides to a full-size product.

We showed that concentrations of oligonucleotides of about 10 nM, Mg^2+^ 10 mM or 2.5 mM spermidine ions are optimal for DNA assembly. The concentrations of oligonucleotides for different polymerases vary slightly. A wide range of concentrations of the oligonucleotides used in the assembly reaction is described: from 2.5 μM [16], 10–0 nM [31], or from 10 nM [32], and the importance of choosing a polymerase is also indicated [32]. The effect of spermidine on the results of DNA assembly is interesting. The addition of spermidine to the reaction mixture avoids an increase in the concentration of magnesium ions in the reaction, and it increases the specificity of the assembly. There are reports of both an improvement in the efficiency of DNA amplification when using spermidine in the reaction [26, 33] and a lack of an effect [34]. At the same time, spermidine promotes the amplification reaction in complex samples [35, 36]. It has been shown that phosphates are the main target in the interaction of spermidine polycation with DNA in B-form [37]. Presumably, spermidine makes it possible for oligonucleotides to hybridize in dsDNA, neutralizing the negative charge of the phosphate backbone and stabilizing duplexes.

Currently, errors in the obtained matrix are a factor limiting *de novo *DNA assembly, which is associated with both the quality of the oligonucleotides and the accuracy of the polymerases [38, 39, 40, 41]. Reducing the number of errors will reduce the complexity that comes with that work and the cost of screening clones and sequencing them to achieve error-free variants. In the case of an optimized technique, the error rate during the assembly of Phusion polymerase was 3 per 1 kb.

We optimized the conditions for combining several DNA fragments 1–1.5 kb long with complementary ends into fragments of up to 7.5 kb long using PCR. The most suitable and widely used methods for combining extended DNA fragments are the Gibson reaction [30] and its variations, as well as homologous recombination in yeast [42, 43]. However, combining multiple amplicons using PCR is a simpler and faster method that uses a smaller variety of enzymes. We showed that a full-size product 2–3 kb long can be obtained under a wide range of conditions common for PCR amplification, and that spermidine does not have a positive effect on reactions of this type, unlike the reaction of DNA assembly from oligonucleotides. With a decrease in temperature and elongation time, fragments of up to 7.5 kb can be combined by amplification.

Thus, in this work, we unified the conditions for the assembly of DNA fragments from oligonucleotides by polymerase chain assembly. We showed that the assembly reaction is efficient at oligonucleotide concentrations in the range of 10 nM, with the addition of 10 mM Mg^2+^ or 2.5 mM spermidine to the reaction mixture. The choice of the oligonucleotide concentration depends on the polymerase. The addition of 2.5 mM spermidine to the reaction mixture makes it possible to increase the specificity of the assembly. The use of a more accurate Phusion polymerase for assembly makes it possible to reduce the number of errors under optimized conditions to 3 per 1 kb, mainly due to fewer substitutions.

To combine DNA fragments obtained from synthetic oligonucleotides, we optimized the conditions for combining several fragments of dsDNA with a size of about 1 kb, with overlapping regions at the ends, into a fragment with a length of up to 7.5 kb.

## References

[1] Agarwal K.L., Büchi H., Caruthers M.H., Gupta N., Khorana H.G., Kleppe K., Kumar A., Ohtsuka E., Rajbhandary U.L., van de Sande J.H. (1970). Nature.

[2] Smith H.O., Hutchison C.A., Pfannkoch C., Venter J.C. (2003). Proc. Natl. Acad. Sci. USA..

[3] Gibson D.G., Benders G.A., Andrews-Pfannkoch C., Denisova E.A., Baden-Tillson H., Zaveri J., Stockwell T.B., Brownley A., Thomas D.W., Algire M.A. (2008). Science..

[4] Venetz J.E., del Medico L., Wölfle A., Schächle P., Bucher Y., Appert D., Tschan F., Flores-Tinoco C.E., van Kooten M., Guennoun R. (2019). Proc. Natl. Acad. Sci. USA..

[5] Annaluru N., Muller H., Mitchell L.A., Ramalingam S., Stracquadanio G., Richardson S.M., Dymond J.S., Kuang Z., Scheifele L.Z., Cooper E.M. (2014). Science..

[6] Lajoie M.J., Rovner A.J., Goodman D.B., Aerni H.R., Haimovich A.D., Kuznetsov G., Mercer J.A., Wang H.H., Carr P.A., Mosberg J.A. (2013). Science..

[7] Chatterjee A., Lajoie M.J., Xiao H., Church G.M., Schultz P.G. (2014). Chembiochem..

[8] Jain K.K. (2013). Med. Princ. Pract..

[9] Peng R.H., Yao Q.H., Xiong A.S., Cheng Z.M., Li Y. (2006). Plant Cell Rep..

[10] Xiong A.S., Yao Q.H., Peng R.H., Zhang Z., Xu F., Liu J.G., Han P.L., Chen J.M. (2006). Appl. Microbiol. Biotechnol..

[11] Casini A., Storch M., Baldwin G.S., Ellis T. (2015). Nat. Rev. Mol. Cell. Biol..

[12] Kosuri S., Church G.M. (2014). Nat. Methods..

[13] Shevelev G.Y., Pyshnyi D. V. (2018). Vavilov J. Genet. Breed..

[14] Dietrich R., Wirsching F., Opitz T., Schwienhorst A. (1998). Biotechnol. Techniques..

[15] Gibson D.G. (2009). Nucleic Acids Research.

[16] Stemmer W.P.C., Crameri A., Ha K.D., Brennan T.M., Heyneker H.L. (1995). Gene..

[17] Xiong A.S., Yao Q.H., Peng R.H., Li X., Fan H.Q., Cheng Z.M., Li Y. (2004). Nucleic Acids Research.

[18] Gao X., Yo P., Keith A., Ragan T.J., Harris T.K. (2003). Nucleic Acids Research.

[19] Sandhu G.S., Aleff R.A., Kline B.C. (1992). Biotechniques..

[20] Xiong A.S., Yao Q.H., Peng R.H., Duan H., Li X., Fan H.Q., Cheng Z.M., Li Y. (2006). Nature Protocols.

[21] Chakrabarti R., Schutt C.E. (2001). Nucleic Acids Research.

[22] Jensen M.A., Fukushima M., Davis R.W. (2010). PLoS One..

[23] Henke W., Herdel K., Jung K., Schnorr D., Loening S.A. (1997). Nucleic Acids Research.

[24] Jurišić V., Obradović J., Tošić N., Pavlović S., Kulić M., Djordjević N. (2016). J. Pharm. Biomed. Anal..

[25] Karunanathie H., Kee P.S., Ng S.F., Kennedy M.A., Chua E.W. (2022). Biochimie..

[26] Wan C.Y., Wilkins T.A. (1993). PCR Methods Appl..

[27] https://sysbiomed.ru/upload/SynthBac.zip.

[28] Garanina I.A., Fisunov G.Y., Govorun V.M. (2018). Front. Microbiol..

[29] Kikin O., D’Antonio L., Bagga P.S. (2006). Nucleic Acids Research.

[30] Gibson D.G., Young L., Chuang R.-Y., Venter J.C., Hutchison C.A., Smith H.O. (2009). Nat. Methods..

[31] Ye H., Huang M.C., Li M.H., Ying J.Y. (2009). Nucleic Acids Research.

[32] Wu G., Wolf J.B., Ibrahim A.F., Vadasz S., Gunasinghe M., Freeland S.J. (2006). J. Biotechnol..

[33] Ahokas H., Erkkilä M.J. (1993). PCR Methods Appl..

[34] Blanchard M.M., Taillon-Miller P., Nowotny P., Nowotny V. (1993). PCR Methods Appl..

[35] Roperch J.P., Benzekri K., Mansour H., Incitti R. (2015). BMC Biotechnol..

[36] Kikuchi A., Sawamura T., Kawase N., Kitajima Y., Yoshida T., Daimaru O., Nakakita T., Itoh S. (2010). Biochem. Genet..

[37] Deng H., Bloomfield V.A., Benevides J.M., Thomas G.J. (2000). Nucleic Acids Research.

[38] Smith H.O., Hutchison C.A., Pfannkoch C., Venter J.C. (2003). Proc. Natl. Acad. Sci. USA..

[39] Sequeira A.F., Brás J.L.A., Guerreiro C.I.P.D., Vincentelli R., Fontes C.M.G.A. (2016). BMC Biotechnol..

[40] Carr P.A., Park J.S., Lee Y.J., Yu T., Zhang S., Jacobson J.M. (2004). Nucleic Acids Research.

[41] Ma S., Saaem I., Tian J. (2012). Trends Biotechnol..

[42] Ma H., Kunes S., Schatz P.J., Botstein D. (1987). Gene..

[43] Gibson D.G., Benders G.A., Axelrod K.C., Zaveri J., Algire M.A., Moodie M., Montague M.G., Venter J.C., Smith H.O., Hutchison C.A. (2008). Proc. Natl. Acad. Sci. USA..

